# Clinical characteristics, laboratory parameters and outcomes of COVID‐19 in cancer and non‐cancer patients from a tertiary Cancer Centre in India

**DOI:** 10.1002/cam4.4379

**Published:** 2021-11-16

**Authors:** Sumeet P. Mirgh, Anant Gokarn, Akhil Rajendra, Ashwini More, Sujit Kamtalwar, Kritika S. Katti, Anuj Singh, Vasu Babu Goli, Rahul Ravind, Ravikrishna Madala, Sangeeta Kakoti, Priyamvada Maitre, Sachin Punatar, Akanksha Chichra, Amar Patil, Bhakti Trivedi, Amit Joshi, Nikhil Patkar, Prashant Tembhare, Twinkle Khanka, Sweta Rajpal, Gaurav Chatterjee, Sadhana Kannan, P.G. Subramanian, Vedang Murthy, Nitin Shetty, Preeti Chavan, Vivek Bhat, Sudhir Nair, Navin Khattry, Sudeep Gupta

**Affiliations:** ^1^ BMT Unit Department of Medical Oncology ACTREC – Tata Memorial Centre Navi Mumbai India; ^2^ Homi Bhabha National Institute Mumbai India; ^3^ Department of Medical Oncology ACTREC – Tata Memorial Centre Navi Mumbai India; ^4^ Department of Medicine ACTREC – Tata Memorial Centre Navi Mumbai India; ^5^ Department of Radiation Oncology ACTREC – Tata Memorial Centre Navi Mumbai India; ^6^ Department of Anaesthesiology, Pain and Critical Care Medicine ACTREC – Tata Memorial Centre Navi Mumbai India; ^7^ Hematopathology Lab Department of Pathology ACTREC – Tata Memorial Centre Navi Mumbai India; ^8^ Department of Biostatistics ACTREC – Tata Memorial Centre Navi Mumbai India; ^9^ Department of Radiodiagnosis ACTREC – Tata Memorial Centre Navi Mumbai India; ^10^ OIC Composite Lab Department of Laboratory Medicine ACTREC – Tata Memorial Centre Navi Mumbai India; ^11^ Department of Microbiology ACTREC – Tata Memorial Centre ACTREC Navi Mumbai India; ^12^ Department of Surgical Oncology ACTREC – Tata Memorial Centre ACTREC Navi Mumbai India

**Keywords:** cancer, COVID‐19, non‐cancer, outcomes

## Abstract

**Background:**

There is paucity of data regarding clinical characteristics, laboratory parameters and outcomes of coronavirus disease (COVID‐19) in cancer versus non‐cancer patients, particularly from India.

**Materials and Methods:**

This was an observational, single‐centre, retrospective analysis of patients with laboratory‐confirmed COVID‐19 hospitalised in our institution between 22 May 2020 and 1 December 2020. We compared baseline clinical characteristics, laboratory parameters and outcomes of COVID‐19 (overall mortality, time to discharge) between cancer and non‐cancer patients.

**Results:**

A total of 200 COVID‐19 infection episodes were analysed of which 109 (54.5%) were patients with cancer and 91 (45.5%) were patients without cancer. The median age was 43 (interquartile range [IQR]:32–57), 51 (IQR: 33–62) and 38 (IQR: 31.5–49.3) years; of whole cohort, cancer and non‐cancer patients, respectively. Comparison of outcomes showed that oxygen requirement (31.2% [95% CI: 22.6–40.7] vs. 17.6% [95% CI: 10.4–26.9]; *p* = 0.03), median time to discharge (11 days [IQR: 6.75–16] vs. 6 days [IQR: 3–9.75]; *p* < 0.001) and mortality (10.0% [95% CI: 5.2–17.3] vs. 1.1% [95% CI: 0.03–5.9]; *p* = 0.017) were significantly higher in patients with cancer. In univariable analysis, factors associated with higher mortality in the whole cohort included diagnosis of cancer (10.1% vs. 1.1%; *p* = 0.027; odds ratio [OR]: 7.04), age ≥60 (17.4% vs. 2.6%; *p* = 0.001; OR: 7.38), oxygen requirement (22% vs. 0.6%; *p* < 0.001; OR: 29.01), chest infiltrates (19.2% vs. 1.4%; *p* < 0.001; OR: 22.65), baseline absolute lymphocyte count <1 × 10^9^/L (10.8% vs. 1.9%; *p* = 0.023; OR:5.1), C‐reactive protein >1 mg% (12.8% vs. 0%; *p* = 0.027; OR: 24.69), serum procalcitonin >0.05 ng/ml (22.65% vs. 0%; *p* = 0.004; OR: 4.49) and interleukin‐6 >6 pg/ml (10.8% vs. 1.3%; *p* = 0.036; OR: 3.08). In multivariable logistic regression, factors significantly associated with mortality were oxygen requirement (*p* = 0.005; OR: 13.11) and high baseline procalcitonin level (*p* = 0.014; OR: 37.6).

**Conclusion:**

Cancer patients with COVID‐19 have higher mortality and require longer hospital stay. High procalcitonin levels and oxygen requirement during admission are other factors that affect outcomes adversely.

## Graphical abstract

This is the first data from India, describing comparison of cancer and non‐cancer patients with COVID‐19 treated with a uniform protocol. It shows that cancer patients with COVID‐19 have higher mortality and require longer hospital stay. High procalcitonin levels and oxygen requirement during admission are factors that adversely affect outcomes.

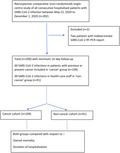

## 1 INTRODUCTION

In the beginning of 2020, a novel RNA coronavirus, named severe acute respiratory syndrome coronavirus (SARS‐CoV‐2), was identified as the causative agent for the pneumonia epidemic affecting the city of Wuhan, in China. Later, World health organisation (WHO) labelled it as coronavirus disease 2019 (COVID‐19) and declared a pandemic. Although majority of cases are asymptomatic or mild, approximately 10% patients have a severe disease.^1^ According to current WHO data, as of 31 August 2021, there have been 216 million cases worldwide, with 2.1% mortality. India alone accounts for nearly 15% cases (32 million) to date, with a case fatality rate of 1.3%.^2^

Initial reports from China suggested that cancer patients are five times more likely to receive mechanical ventilation, and succumb to COVID‐19, with mortality rates approaching as high as 28%.^3^, ^4^ In contrast, Brar et al,^5^ interestingly, did not find any significant difference in morbidity and mortality of cancer versus non‐cancer patients with COVID‐19. With respect to India, one study in children with cancer did not report any mortality with COVID‐19, whereas three adult studies reported variable mortality rates between 10% and 20%.^6^, ^7^, ^8^, ^9^ Zhang et al, observed that cancer patients who had received anti‐cancer therapy within 2 weeks of contracting SARS‐CoV‐2, were at higher risk of developing severe COVID‐19 and mortality. Hence, they advocated avoiding or reducing doses of immunosuppressive medications for cancer patients,^4^ which was later contradicted by Brar et al.^5^ Compromising on malignancy treatment might be detrimental for cancer in long‐term. In view of the above conflicting results,^3^, ^4^, ^5^ absence of a cohort of non‐cancer patients as a control arm for comparison, treatment with non‐uniform policies which evolved over time,^3^, ^4^, ^6^, ^7^, ^8^, ^9^ and absence of any data from India comparing cancer versus non‐cancer patients, we conducted this retrospective analysis to compare differences in clinical presentation, laboratory parameters and outcomes of cancer and non‐cancer patients in a tertiary centre.

## 2 METHODS

**FIGURE 1:**
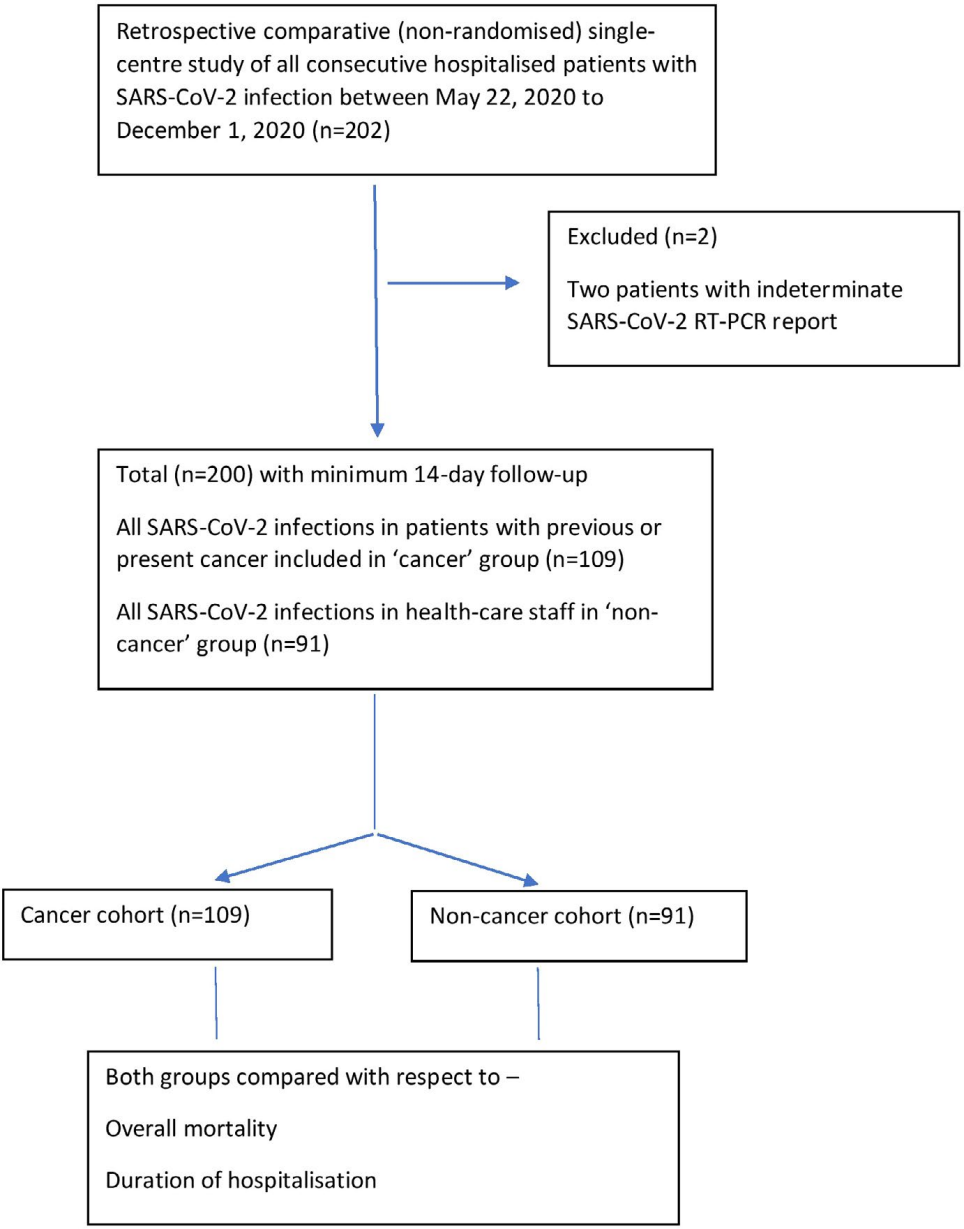
CONSORT diagram of the study

This was a single‐centre, retrospective study from a tertiary cancer centre in India. All consecutive hospitalised patients diagnosed with SARS‐CoV‐2 infection between 22 May 2020 and 1 December 2020 were included (Figure 1). Patients with both haematological and solid organ malignancies comprised the cancer cohort, whereas the non‐cancer cohort comprised health care workers of the institute. SARS‐CoV‐2 infection was diagnosed based on quantitative real‐time reverse transcriptase‐polymerase chain reaction (qRT‐PCR) of nasal and/or oropharyngeal swabs. A minimum follow‐up of at least 14 days was required from the first positive swab test. Any patient with less than 14‐day follow‐up or COVID‐19 suspected only on the basis of radiological criteria or with indeterminate SARS‐CoV‐2 RT‐PCR results were excluded.

Baseline demographic data including comorbidities, cancer type, symptoms (including date of onset), date of SARS‐CoV‐2 positivity, date of admission, reason for admission, severity of illness, cancer remission status and ongoing therapy for cancer were extracted from electronic medical health records. For those patients with diagnosis of cancer, any chemotherapy or radiotherapy or major surgery within 4 weeks of SARS‐CoV‐2 positivity were grouped in ‘active anti‐cancer treatment’ cohort. Remission of the malignant disease was defined as absence of disease at last follow‐up before contracting SARS‐CoV‐2 infection.

### 2.1 COVID‐19 management

COVID‐19 severity was graded as per 7‐point ordinal scale published by Goldman et al.^10^ Since we included only hospitalised patients, only those with scores 1–6 were included in this analysis. Patients on admission underwent routine blood tests––hemogram, liver/renal function tests with electrolytes, coagulation profile, fibrinogen (laboratory normal: 150–400 mg/dl), D‐Dimer (laboratory normal <200 ng/ml), inflammatory markers (C‐reactive protein [CRP] [laboratory normal <0.3 mg%], procalcitonin [PCT] [laboratory normal <0.05 ng/ml] and interleukin‐6 [IL‐6] [laboratory normal <6 pg/ml]), lymphocyte subset analysis (CD3, CD4, CD8, B, natural killer [NK] cells and T regulatory cells [Treg]) and imaging (chest X‐ray or computerised tomogram [CT] of thorax). Blood tests were repeated every 96 h, every 48 h and every 24 h for patients with very mild, mild and moderate‐severe disease (as per ordinal scale), respectively. IL‐6 was performed at baseline and prior to administration of tocilizumab. Lymphocyte subset analysis was performed at baseline and weekly, until discharge. Chest X‐rays were repeated every 96 h for patients with moderate‐severe disease, and as per physician discretion.

Patients with moderate‐severe disease (ordinal scale score: 2–4) received antivirals (5‐day remdesivir or a combination of lopinavir/ritonavir [LPV/r] + ribavirin [RBV] ± interferon‐β [IFN‐β]‐1a [prior to availability of remdesivir in India]), oxygen support and anti‐inflammatory agents (dexamethasone and/or tocilizumab). Patients with mild (score 5) or very mild disease (score 6) received symptomatic supportive care. However, mild COVID‐19 patients (score 5) received antivirals, if they were at high risk (post‐hematopoietic stem cell transplant [HSCT] or within 1‐month post‐chemotherapy or absolute lymphocyte count (ALC) <0.6 × 10^9^/L at admission) or those with persistent fever (temperature >101°F for >48 h) or tachypnoea (respiratory rate >24 per minute) with or without elevated inflammatory markers (CRP >5 mg%). All those who received antivirals received prophylactic low‐molecular weight heparin (1 mg/kg/day enoxaparin subcutaneously), except those with platelet count <50 × 10^9^/L. Remdesivir was given for 5 days, as per Goldman et al^10^––200 mg intravenously on Day 1, followed by 100 mg intravenously for subsequent 4 days. LPV/r (400/100 mg) and RBV (400 mg) were given twice daily for a duration of 14 days as per Hung et al.^11^ Dexamethasone was variably dosed between 6 and 10 mg as per RECOVERY^12^ and CoDEX^13^ trials for a maximum duration of 10 days. Tocilizumab was administered at a dose of 400 mg, amounting to a variable dose of 4–8 mg/kg.^14^ A second dose was repeated after 12–24 h, at the discretion of the treating physician.

Patients were discharged according to national guidelines, which entailed discharge only after 10 days of symptom onset, with at least 3 days of asymptomatic period. Negative swab was not required prior to discharge.^15^ The decision for repeating nasal/oropharyngeal swabs for SARS‐CoV‐2 has been dynamic, and evolved over time. Initially, swabs were repeated every week, until negativity. However, since majority of our patients continued to be positive until Day 22, we amended (after 50 patients) our institutional policy to perform a second swab on Day 22 after first SARS‐CoV‐2 RT‐PCR positivity, along with testing for SARS‐CoV‐2 antibodies on Day 22. Those who were SARS‐CoV‐2 RT‐PCR positive on Day 22, were swabbed every week until a negative result was obtained.

The primary endpoint of the study was overall mortality between cancer versus non‐cancer patients. It was calculated from first date of SARS‐CoV‐2 RT‐PCR positivity until date of death during hospital stay. The secondary endpoints were duration of hospitalisation in cancer and non‐cancer patients, and factors affecting mortality. Duration of hospitalisation was calculated as duration (days) from date of admission until date of discharge or date of death, if death occurred during hospital stay.

### 2.2 Statistical analysis

Categorical variables were expressed as frequencies (with percentages). Continuous variables were represented as median (with interquartile range [IQR]). The Fisher's exact test was used to compare categorical data and the Mann–Whitney *U* test for comparing continuous data. All the statistical analyses were performed using spss version 23.0. For univariable and multivariable analyses, due to low number of events a penalised maximum likelihood estimator for logistic regression model using Firth correction was used to identify factors associated with mortality. Reported *p* values were two‐sided and a *p* value of <0.05 was considered statistically significant.

## 3 RESULTS

### 3.1 Cohort characteristics

A total of 198 consecutive hospitalised patients with 200 SARS‐CoV‐2 infection episodes (two patients with re‐infection) were analysed. Characteristics of patients, both with and without cancer are shown in Table 1. Of these, 109 episodes were in cancer patients and remaining 91 in non‐cancer patients. Median age of the whole cohort was 43 years (IQR: 32–57), with a male predominance (63%). Median age was higher in cancer cohort (*p* < 0.001). Twenty‐three per cent (*n* = 46) of all patients were ≥60 years, and 5% (*n* = 10) were under 18 years.

**TABLE 1:** Baseline clinical characteristics

	Patients with cancer (cancer cohort), *N* = 109 (54.5%)	Health care workers (non‐cancer cohort), *N* = 91 (45.5%)	All patients, *N* = 200 (100%)	*p* value
Median age (years)	51	38	43	<0.001
Interquartile range (IQR)	33–62	32–49	32–57	
Gender				
Male	64 (58.7)	62 (68.1)	126 (63)	NS
Female	45 (41.3)	29 (31.9)	74 (37)	
Comorbidities	29 (26.6)	37 (40.6)	66 (33)	0.035
Diabetes mellitus	9 (8.1)	13 (14.6)	22 (11)	
Hypertension	19 (17.1)	21 (23.6)	40 (20)	
Others	1 (0.9)	3 (3.3)	4 (2)	
Symptoms (*n* = 200)				
Fever	62 (56.9)	66 (72.5)	128 (64)	0.02
Cough	41 (37.7)	53 (58.2)	94 (47)	0.003
Breathlessness (all grades)	31 (28.4)	25 (27.4)	56 (28)	NS
Severe breathlessness (NYHA 3–4)	18 (16.5)	6 (6.5)	24 (12)	0.05
Sore throat	18 (16.5)	28 (30.8)	46 (23)	0.017
Fatigue	27 (24.7)	43 (47.2)	70 (35)	<0.001
Myalgia	18 (16.5)	43 (47.3)	61 (30.5)	<0.001
Headache	14 (12.8)	36 (39.5)	50 (25)	<0.001
Nausea	12 (11)	20 (22)	32 (16)	0.035
Vomiting	6 (5.5)	9 (9.9)	15 (7.5)	NS
Loss of taste	7 (6.4)	20 (21.9)	27 (13.5)	0.001
Loss of smell	2 (1.8)	16 (17.5)	18 (9)	<0.001
Rhinorrhoea	7 (6.4)	13 (14.3)	20 (10)	0.06
Odynophagia	5 (4.6)	5 (5.5)	10 (5)	NS
Conjunctival congestion	0 (0)	3 (3.2)	3 (1.5)	NS
Median symptom duration prior to admission in days (IQR)	3 (2–5)	3 (2–5)	3 (2–5)	NS
Baseline COVID‐19 severity, as per ordinal scale [1]				
Very mild (Score 6)	23 (21.1)	33 (36.2)	56 (28)	
Mild (score 5)	61 (55.9)	47 (51.6)	107 (53.5)	
Moderate (score 4)	13 (11.9)	9 (9.9)	22 (11)	
Severe (score 2, 3)	12 (11)	2 (2.2)	15 (7.5)	
COVID−19 infection requiring oxygen, at admission	25 (22.9)	11 (12.1)	36 (18)	0.06
Baseline chest X‐ray				
Available	105 (96.3)	88 (96.7)	193 (96.5)	0.001
Normal chest X‐ray	66 (62.8)	73 (82.9)	139 (72)	
Abnormal chest X‐ray	39 (37.2)	15 (17.1)	54 (27.9)	
Left infiltrates	17 (16.2)	8 (9)	25 (12.9)	
Right infiltrates	3 (2.8)	1 (1.1)	4 (2.07)	
Bilateral	19 (18)	6 (6.8)	25 (12.9)	

Abbreviations: COVID‐19, coronavirus disease; IQR, interquartile range; NS, not significant; NYHA, New York Heart Association.

Amongst cancer patients, 55.9% (*n* = 61) were afflicted with solid organ tumours (head and neck [*n* = 12; 11%], breast [*n* = 18; 16.5%], lung [*n* = 4; 3.5%], gastrointestinal [*n* = 8; 7.3%], genitourinary [*n* = 11; 10%], bone/soft tissue [*n* = 7; 6.4%] and carcinoma of unknown primary [*n* = 1; 0.9%] and remaining had haematological malignancies [*n* = 48; 44.1%] [acute leukaemia [*n* = 11; 10.1%], lymphoma [*n* = 16; 14.7%], chronic lymphocytic leukaemia [*n* = 3; 2.8%], myeloproliferative neoplasm [*n* = 7; 6.4%], multiple myeloma [*n* = 10; 9.2%] and myelodysplastic syndrome [*n* = 1; 0.9%]). Nearly half of the cancer patients (*n* = 51; 46.8%) were in remission at time of infection. Amongst cancer patients, 72.5% (*n* = 79) received chemotherapy in the preceding 1 month (active treatment) of SARS‐CoV‐2 positivity. Amongst patients of haematological malignancies, nine (18.75%) received rituximab within 6 months of SARS‐CoV‐2 infection. Nine (8.2%) cancer patients had undergone surgery and seven (6.4%) received radiotherapy within 4 weeks of acquiring SARS‐CoV‐2 infection.

### 3.2 COVID‐19––baseline characteristics

Most symptoms (Table 1) were more common in non‐cancer than in cancer patients, except severe breathlessness at presentation (grade 3 and 4 as per New York Heart Association [NYHA]) (16.5% [95% CI: 10.1–24.8] vs. 6.5% [95% CI: 2.4–13.8]; *p* = 0.05), tachypnoea (respiratory rate >24 per minute) (23.8% [95% CI: 16.2–33] vs. 10.9% [95% CI: 5.4–19.3]; *p* = 0.018), adventitious sounds on auscultation (15.6% [95% CI: 9.3–23.8] vs. 4.3% [95% CI: 1.2–10.9; *p* = 0.019]), lung infiltrates on radiology (37.1% [95% CI: 26.8–45.5] vs. 17% [95% CI: 9.5–25.7]; *p* = 0.001) and oxygen support at admission (22.9% [95% CI: 15.4–32] vs. 12.1% [95% CI: 6.2–20.6]; *p* = 0.06).

Baseline laboratory characteristics at admission (Table 2) show that patients with cancer had significantly higher incidence of anaemia (*p* < 0.001) (anaemia defined as hemoglobin <13 g/dl for males and <12 g/dl for females),^16^ leukocytosis (total leukocyte count >10 × 10^9^/L) (*p* < 0.001) and elevated D‐dimers (>200 ng/ml) (*p* = 0.06), in comparison to non‐cancer patients. The median ALC and lymphocyte subsets (CD3, CD4, CD8, Treg, NK and B cells) were significantly lower in cancer patients, while median fibrinogen levels (*p* < 0.001) and IL‐6 (*p* < 0.001) were significantly higher in cancer cohort. While median CRP was elevated in cancer cohort (*p* < 0.001), the median PCT levels were similar in both groups.

**TABLE 2:** Baseline laboratory characteristics

Variables	Patients with cancer (cancer cohort), *N* = 109	Health care workers (non‐cancer cohort), *N* = 91	All patients (*N* = 200)	*p* value
CBC parameters (n = 196); Median (IQR)				
Hemoglobin	10.4 (9–12)	13.6 (12.4–14.6)	12 (9.8–13.8)	<0.001
TLC (×10^9^/L)	5.83 (2.69–9.6)	5.26 (4.1–6.8)	5.37 (3.6–8.05)	NS
ANC	3.5 (1.6–6.2)	3.2 (2.2–4.5)	3.3 (1.9–5.1)	NS
ALC (×10^9^/L)	0.84 (0.42–1.32)	1.39 (0.86–1.79)	1.075 (0.65–1.62)	<0.001
Platelet (×10^9^/L)	225 (113–336)	213 (168.5–278.5)	221.5 (153–300)	NS
CBC parameters (*n* = 196), *n* (%)				
TLC >10(×10^9^/L)	18 (16.5)	2 (2.2)	20 (10)	<0.001
TLC <4(×10^9^/L)	36 (33)	21 (23)	57 (23.5)	NS
ANC <1(×10^9^/L)	16 (8)	0	16 (8)	0
ALC <1(x10^9^/L)	61 (55.9)	32 (35.1)	93 (46.5)	0.05
Inflammatory markers				
Median CRP (IQR); (mg%) (*n* = 194)	2.3 (0.7–7.97)	0.4 (0.3–1.0)	0.9 (0.3–4.5)	<0.001
Median IL‐6 (IQR); (pg/ml) (n = 178)	11 (5–34)	4 (2–9)	7 (3–19)	<0.001
Median Procalcitonin (IQR); (ng/ml) (*n* = 168)	0.05 (0.05–0.19)	0.05 (0.05–0.05)	0.05 (0.05–0.09)	NS
Coagulation parameters				
Median fibrinogen (IQR); (mg/dl) (*n* = 172)	382.5 (304.5–467.75)	301.5 (250.75–381.75)	344 (265–442.5)	<0.001
D‐Dimer (ng/ml) (n = 174), *n* (%)				
Normal (<200)	42 (45.1)	48 (59.2)	90 (51.7)	
Abnormal (>200)	51 (54.8)	33 (40.70)	84 (48.2)	0.06
Lymphocyte subset analysis; median (IQR) (*n* = 170)				
CD3%	68.48 (55.6–76.9)	67.82 (62.1–75.03)	68.29 (59.8–75.8)	NS
CD3 (×10^9^/L)	0.54 (0.27–0.93)	0.87 (0.62–1.21)	0.71 (0.41–1.13)	<0.001
CD4 (×10^9^/L)	0.26 (0.14–0.49)	0.54 (0.35–0.72)	0.37 (0.19–0.64)	<0.001
CD8 (×10^9^/L)	0.21 (0.11–0.38)	0.28 (0.21–0.4)	0.26 (0.15–0.39)	0.025
Treg (×10^9^/L)	0.02 (0.01–0.04)	0.04 (0.03–0.05)	0.03 (0.01–0.05)	<0.001
NK (×10^9^/L)	0.1 (0.05–0.21)	0.13 (0.09–0.25)	0.12 (0.06–0.21)	0.007
B (×10^9^/L)	0.08 (0.015–0.24)	0.18 (0.11–0.28)	0.14 (0.05–0.27)	<0.001

### 3.3 COVID‐19 treatment during hospitalisation

Median time to worst COVID‐19 score was 1 day after SARS‐CoV‐2 positivity in both groups. Apart from 36 patients (cancer [*n* =25] and non‐cancer [*n* =11]) who required oxygen support at baseline, 9 (10.8%) and 5 (6.3%) patients in cancer and non‐cancer subgroup, respectively, required initiation of oxygen support due to clinical worsening during hospitalisation. As shown in Table 3, significantly more patients with cancer required treatment with antivirals (54.2% [95% CI: 44.3–63.7] vs. 28.5% [95% CI: 19.6–39]; *p* = 0.002), oxygen support (31.2% [95% CI: 22.6–40.7] vs. 17.6% [95% CI: 10.4–26.9]; *p* = 0.033) and longer duration of oxygen support (days) (6 vs. 3.5; *p* = 0.08).

**TABLE 3:** Treatment characteristics during admission

Variables	Patients with cancer (cancer cohort), *N* = 109	Health care workers (non‐cancer cohort), *N* = 91	All patients (*N* = 200)	*P* value
Median follow‐up duration in days (IQR)	22 (15–42)	22 (20–30)	22 (17–37)	NS
Treatment received; n (%)				
Antivirals	59 (54.2)	26 (28.5)	85 (42.5)	0<0.001
LPV/r + RBV +IFN‐β1b	11 (10.1)	4 (4.4)	15 (7.5)	
Remdesivir	48 (44)	22 (24.2)	70 (35)	
Anti‐inflammatory	26 (23.8)	14 (15.4)	40 (20)	NS
Dexamethasone alone	13 (11.9)	9 (9.9)	22 (11)	
TCZ alone	8 (7.3)	2 (2.2)	10 (5)	
TCZ +Dexamethasone	5 (4.6)	3 (3.3)	8 (4)	
LMWH	54 (49.5)	27 (29.7)	81 (40.5)	0.004
Median LMWH duration in days (IQR)	10 (7–14)	8 (5–12)	9 (7–14)	0.09
TCZ use, n (%)	13 (11.9)	5 (5.4)	18 (9)	NS
Dose of TCZ; n (%)				
4 mg/kg	2 (1.8)	0	2 (1)	NS
6 mg/kg	5 (4.6)	2 (2.2)	6 (3)	
8 mg/kg	6 (5.5)	3 (3.3)	10 (5)	
Oxygen support during hospitalisation, n (%)				
Yes	34 (31.2)	16 (17.6)	50 (25)	0.033
No	75 (68.8)	75 (82.4)	150 (75)	
Median duration of oxygen support in days (IQR)	6 (2–9.5)	3.5 (1–5)	5 (2–8)	0.08
Median duration of hospitalisation in days (IQR)	11 (6.75–16)	6 (3–9.75)	8 (5–14)	<0.001

### 3.4 Outcomes of COVID‐19

Median follow‐up duration was similar in both cohorts (22 days; IQR: 17–37). There was significantly higher overall mortality (10.1% [95% CI: 5.2–17.3] vs. 1.1% [95% CI: 0.03–5.9]; *p* = 0.017) in cancer versus non‐cancer patients. In deceased patients with cancer, every patient except one, died within 28 days of diagnosis of SARS‐CoV‐2 infection. Importantly, median duration of hospital stay was much longer in cancer (11 days; IQR: 6.75–16) versus non‐cancer cohort (6 days; IQR: 3–9.75) (*p* < 0.001). Although equivalent proportion of subjects in both groups (*n* =48; 55.2% [95% CI: 44.1–65.8] vs. *n* = 38; 51.3% [95% CI: 39.4–63.2]; *p* = 0.62) became COVID‐19 RT‐PCR negative on Day 22, higher proportion of non‐cancer patients (*n* = 48; 64.9% [95% CI: 52.9–72.6]) developed SARS‐CoV‐2 antibodies (at Day 22), in comparison to cancer (*n* = 34; 36.2% [95% CI: 26.5–46.7]) patients (*p* < 0.001).

On univariable analysis (Table 4), presence of cancer (*p* = 0.027), age ≥60 years (*p* = 0.001), need of oxygen during hospitalisation (*p* < 0.001), infiltrates on X‐ray (*p* < 0.001), baseline ALC<1 × 10^9^/L (*p* = 0.023), baseline CRP>1 mg% (*p* = 0.027), abnormal baseline PCT > 0.05 ng/ml (*p* = 0.004) and elevated IL‐6 >6 pg/ml (*p* = 0.036) were found to be adversely associated with increased mortality. However, on multivariable analysis, need of oxygen supplementation (*p* = 0.005) and abnormal PCT (*p* = 0.014) were factors that adversely affected mortality. In cancer patients’ cohort (Table 5), presence of active malignancy (i.e. cancer not in remission) (*p* = 0.01), need of oxygen (*p* = 0.001) and abnormal PCT (*p* = 0.026) were significant factors affecting mortality both on univariable and multivariable analyses. Receipt of active anti‐cancer treatment within the preceding month of SARS‐CoV‐2 positivity was not associated with death in cancer patients.

**TABLE 4:** Univariable analysis of factors affecting death, both in overall and cancer population

Whole cohort (*n* = 200)	No of deaths/total	Odds ratio	95% CI	*p* value
Cancer	11/109	7.04	1.25–39.54	0.027
Non‐cancer	1/91	Ref	—	—
Age (years)				
≥60	8/46	7.38	2.23–24.42	0.001
<60	4/154	Ref	—	—
Oxygen support				
Yes	11/50	29.01	5.11–164.86	<0.001
No	1/150	Ref	—	—
Baseline chest X‐ray				
Abnormal	10/52	22.65	3.95–129.68	<0.001
Normal	2/148	Ref	—	
Baseline ALC (×10^9^/L)				
≤1	10/93	5.1	1.25–20.90	0.023
>1	2/103	Ref	—	—
Baseline CRP (mg%)				
>1	12/94	24.69	1.44–424.07	0.027
≤1	0/81	Ref	—	—
Baseline PCT (ng/ml)				
>0.05	12/53	4.49	2.19–9.18	0.004
≤0.05	0/115	Ref	—	—
Baseline IL‐6 (pg/ml)				
>6	11/102	3.08	1.49–6.36	0.036
≤6	1/76	Ref	—	—
Cancer cohort (*n* = 109)				
Cancer in remission				
Yes	10/61	6.46	1.11–37.36	0.037
No	1/48	Ref	—	—
Oxygen support				
Yes	10/34	21.28	3.62–125.04	0.001
No	1/75	Ref	—	—
Active chemotherapy				
Yes	9/82	1.32	0.30–5.70	0.711
No	2/27	Ref	—	—
Baseline CRP (mg%)				
>2.5	10/52	8.65	1.49–50.16	0.016
≤2.5	1/53	Ref	—	—
Baseline PCT (ng/ml)				
>0.05	11/40	39.37	2.24–692.77	0.012
≤0.05	0/50	Ref	—	—
Baseline IL‐6 (pg/ml)				
>11	9/46	5.22	1.22–22.37	0.026
≤11	2/53	Ref	—	—
Baseline IL‐6 (pg/ml)				
>50	6/23	4.82	1.38–16.85	0.014
≤50	5/76	Ref	—	—

**TABLE 5:** Multivariable analysis of factors affecting death, both in overall and cancer population

Factors	Odds ratio	95% CI	*p* value
Whole cohort (*n* = 200)			
Oxygen support			
Yes	13.11	2.15–80.09	0.005
No	Ref	—	—
Baseline PCT (ng/ml)			
>0.05	37.6	2.11–671.98	0.014
≤0.05	Ref	—	—
Cancer cohort (*n* = 109)			
Cancer in remission			
Yes	18.47	1.99–171.02	0.01
No	Ref	—	—
Oxygen support			
Yes	35.68	4.09–311.32	0.001
No	Ref	—	—
Baseline PCT (ng/ml)			
>0.05	35.46	1.53–824.11	0.026
≤0.05	Ref	—	—

## 4 DISCUSSION

This single‐centre retrospective study provides detailed information on clinical characteristics, laboratory parameters and outcomes in Indian patients with or without cancer afflicted with SARS‐CoV‐2 infection treated with a uniform protocol. Our cohort consisted of 109 cancer and 91 non‐cancer SARS‐CoV‐2 infections, respectively.

The median age of our cancer (51 years) and non‐cancer cohorts (38 years) was similar to other Indian data,^9^, ^17^ but nearly three decades earlier than reported from the Western literature of COVID‐19 in cancer.^5^, ^18^, ^19^, ^20^ However, in contrast to another Indian study by Ramaswamy et al,^9^ we had a greater proportion of elderly cancer patients (>60 years) (23% vs. 14%). One‐fourth of our cancer patients had moderate‐severe disease (*n* = 50; 25%) at admission or during hospital stay, higher than reported by Ramaswamy et al.^9^ (15%), however, significantly lesser than other Indian (48%)^8^ and Western data (52%–55%).^5^, ^18^ Importantly, the majority of our cancer patients (79%) were on active anti‐cancer treatment at the time of SARS‐CoV‐2 positivity, in contrast to others (22%‐60%).^5^, ^7^, ^18^ Also, half of our cancer patients’ malignancies were not in remission (53.2%), much higher than other Indian data (14%–26%).^7^, ^9^

With respect to symptomatology, frequency of fever, cough and constitutional symptoms (fatigue, myalgia and headache) were less common in our malignancy cohort, unlike another report of cancer versus non‐cancer patients.^5^ As opposed to Brar et al.^5^ which reported equivalent percentages in both cohorts, double the number of cancer patients in our study presented with infiltrates on imaging (37.2% vs. 17.1%) and oxygen requirement at admission (22.9% vs. 12.1%). In contrast to Western literature,^5^ our cancer patients demonstrated significant lymphopenia and elevated inflammatory markers (CRP and IL‐6) versus non‐cancer patients. There is a paucity of literature on various lymphocyte subsets in cancer patients with COVID‐19. Similar to data from China^21^ and France,^22^ our cohort showed significantly reduced CD3 and CD4 lymphocytes in cancer subjects. However, other lymphocyte subsets such as NK, B, CD8 T lymphocytes and T regulatory cells were also significantly reduced in our cancer cohort. Whether reduction of all lymphocyte subsets is due to the effect of ongoing or preceding chemotherapy, underlying cancer or specific to SARS‐CoV‐2 infection in cancer patients is unknown. Ours is the first study from India presenting all laboratory parameters and lymphocyte subsets, in cancer and non‐cancer patients.

In contrast to the Indian literature on COVID‐19 in cancer,^7^, ^9^ half of our cancer patients received antivirals (54.2%). Our antiviral policy was uniform, either LPV/r + RBV ± IFN‐β‐1b or remdesivir, unlike other data from India^7^, ^8^, ^9^ and USA.^5^ A higher proportion of our cancer (vs. non‐cancer) patients needed oxygen (31.2% vs. 17.6%), antivirals (54.2% vs. 28.5%) and anti‐inflammatory agents (23.8% vs. 15.4%), contrary to Brar et al who did not report any difference.^5^ This may be due to an elderly patient cohort (median age—71 years) with a similar proportion of patients with moderate‐severe disease in both groups, in their study.^5^ Importantly, in our study, cancer patients required twice the duration of oxygen support (6 vs. 3.5 days), in comparison to their non‐cancer counterparts.

A meta‐analysis of >46,000 patients (cancer patients = 1776) showed that all‐cause mortality was higher in cancer patients with a relative risk of 1.66, as compared to non‐cancer patients. However, none of the studies included in the meta‐analysis were from India.^23^ Notably, mortality rate of 10% in our cancer cohort is much less than what is reported from the Western world (22.5%–28%) and from other Indian studies (20%).^8^ This might be due to a younger population (43 vs. 69–76 years) and lesser proportion of patients with comorbidities (26.6% vs. 40%–75%) in our cohort versus others.^5^, ^8^, ^18^, ^20^ Similar to Ruthrich et al,^18^ we observed a significant difference in mortality rates of cancer (10.1%) versus non‐cancer patients (1.1%). However, this is in contrast to other Western data.^5^, ^24^ Similar to others, need of oxygen (reflection of moderate‐severe disease),^7^, ^8^, ^9^ higher age,^5^, ^19^, ^20^, ^25^ presence of infiltrates on imaging^5^ and absence of remission for cancers^8^, ^9^, ^19^, ^25^ predicted poor survival in our cohort. However, our study is the first to show that an abnormal PCT (>0.05 ng/ml) and elevated CRP (>1 mg% in whole cohort, >2.5 mg% in cancer cohort) predicts worse outcome. In contrast to others, we could not find an increased risk of mortality with gender (male sex)^18^, ^19^ or type of malignancy.^26^ Unlike initial data from China^4^ but similar to recent literature,^7^, ^8^, ^20^ there was no association of active anti‐cancer therapy with poor outcomes in our cohort.

Limitations of our study include a retrospective data set, heterogeneous patient cohort with respect to COVID‐19 severity and inclusion of only hospitalised patients, thereby reducing its applicability in outpatient setting. Since overall mortality included all‐cause mortality during hospitalisation, COVID‐19 attributable mortality cannot be discerned. However, a uniform treatment protocol for both cancer and non‐cancer patients from a single institution and detailed analysis of laboratory parameters constitute our strengths. Our findings imply that cancer patients with COVID‐19 are a high‐risk cohort who require proactive strategies like early hospitalisation with close monitoring and should therefore be on priority list of vaccination policy of various nations.

## 5 CONCLUSION

Cancer patients with COVID‐19 have higher mortality and require longer hospital stay. Higher procalcitonin levels at admission and oxygen requirement during admission are other factors that affect outcomes adversely.

## CONFLICT OF INTEREST

None.

## 6 Author contribution

SM, AG, AR, AM, SK, KK collected information; SM wrote the first draft of the manuscript; SM, AG, AR, AM, SK, AS, SK, PM, VG, RR, RM, AP, BT, AJ, NK were involved in patient management; SM, AG, SK, SP, PGS, VM, NK, SG critically reviewed the manuscript; NP, PT, PGS, TK, SR, GC, PC, VB were involved in laboratory evaluation of the patient; NS reported the radiological findings of the patient; SM, AG, SK did statistical analysis of data; SM and NK finalized the manuscript.

## ETHICAL STATEMENT

Approval obtained from Institutional Ethics Committee (Project #900864, dated 26.08.2021). All clinical investigations detailed in manuscripts submitted to the journal are conducted in accordance with the Declaration of Helsinki.

## Data Availability

Data available upon request from the authors.
